# Treatment of steroid‐resistant checkpoint inhibitor pneumonitis with pirfenidone: A case report

**DOI:** 10.1111/1759-7714.13921

**Published:** 2021-06-18

**Authors:** Kang Miao, Yan Xu, Wenshuai Xu, Ying Zhang, Yongjian Xu, Xinlun Tian, Li Zhang

**Affiliations:** ^1^ Department of Pulmonary and Critical Care Medicine Peking Union Medical Hospital, Chinese Academy of Medical Science & Peking Union Medical College Beijing China

**Keywords:** case report, checkpoint inhibitor pneumonitis, pirfenidone, steroid‐resistance

## Abstract

With the increased use of immune checkpoint inhibitors (ICIs) in lung cancer, which are of great benefit to patients, more and more immune‐related adverse events (irAEs) are being reported. Checkpoint inhibitor pneumonitis (CIP) is one of the most challenging adverse events, which pose a huge challenge to clinical diagnosis and treatment, and its incidence in the real world is greatly underestimated. Currently, the treatment of CIP mainly depends on the use of glucocorticoids. As for steroid‐resistant CIP, there is no unified standardized treatment strategy. Herein, we report a case of steroid‐resistant CIP induced by pembrolizumab in a patient with advanced non‐small cell lung cancer (NSCLC), in which their symptoms were successfully controlled with pirfenidone.

## Graphical abstract

Herein, we report a case of steroid‐resistance checkpoint inhibitor pneumonitis induced by pembrolizumab in advanced non‐small cell lung cancer, and the patient's symptoms were successfully controlled by pirfenidone.

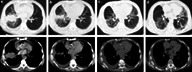

## INTRODUCTION

The use of immune checkpoint inhibitors (ICIs) in lung cancer has provided new hope for patients with advanced lung cancer.^1^ However, ICIs may induce corresponding toxic reactions to normal tissue, collectively known as immune‐related adverse events (irAEs), while mediating tumor immunity.^2^

Among the various AEs, checkpoint inhibitor pneumonitis (CIP) is a class of adverse event that presents a great challenge to clinical diagnosis and treatment. It has previously been defined as newly developed pulmonary infiltrating inflammation (on chest imaging) with exacerbated dyspnea after receiving ICI treatment, under the premise of excluding lung infection or tumor progression.^3^

## CASE REPORT

**FIGURE 1:**
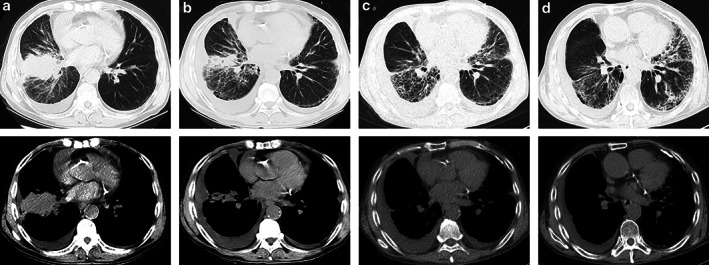
Chest computed tomography (CT) scan. (a) Before starting treatment, (b) After the first course of pemetrexed and carboplatin (AC) + pembrolizumab treatment. (c) After the second course of AC + pembrolizumab treatment; (d) After three weeks of steroid therapy

The patient was a 69‐year‐old man in which chest computed tomography (CT) revealed mass shadows near the right hilum, multiple lymph node metastases in the right supraclavicular region and mediastinum, right pleural and pericardial effusions (Figure 1(a)). Pathological biopsy revealed lung adenocarcinoma, and next generation sequencing (NGS) confirmed that *EGFR*, *ALK*, *KRAS*, *HER‐2*, *BRAF* and *ROS‐1* mutations were all negative with a PD‐L1 expression < 1%. The final diagnosis was adenocarcinoma of the right lung (T2N3M1a, stage IVa).

**FIGURE 2:**
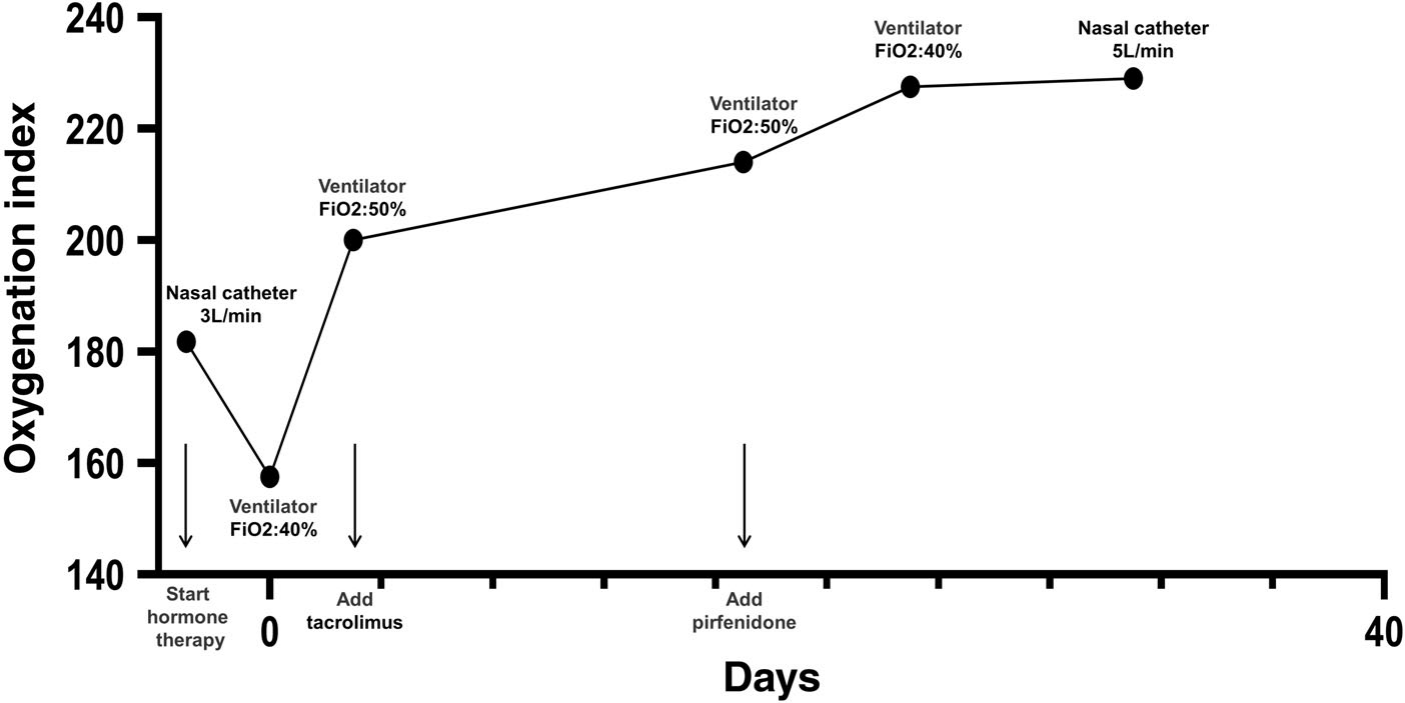
Oxygenation index: Day 0 was recorded as the beginning of noninvasive mechanical ventilation

The patient subsequently received a first course of pemetrexed and carboplatin (AC) + pembrolizumab treatment. After the first course of combined treatment, slight shortness of breath and cough without fever was evident. Chest CT showed that the primary lesion in the right lung and most of the lymph nodes had shrunk, but new ground‐glass opacities (GGOs) were visible in the right middle and lower lobe with increased hydrothorax (Figure 1(b)). The patient subsequently received empirical anti‐infective therapy and a second course of AC + pembrolizumab treatment. Seven days after the second course of combined treatment, the patient's shortness of breath was significantly aggravated, and chest CT re‐examination showed that the range of interstitial pneumonitis had increased, accompanied by extensive new interstitial inflammation and pericardial effusion (Figure 1(c)). Considering there was had been no obvious curative effect with anti‐infective therapy, no positive findings on infection‐related examinations, such as bacterial smear and culture, and BNP levels had improved rapidly after diuretic treatment, we considered the patient to have CIP. We therefore ceased treatment with pembrolizumab, and administered methylprednisolone (100 mg/12 h) by intravenous injection, together with immunoglobulin and cefoperazone. After three days of combined treatment, the symptoms were further aggravated and the patient was admitted to the emergency room. Noninvasive ventilator assisted ventilation (IP 12 cmH_2_O, EP 5 cmH_2_O, FiO_2_ 40%) was commenced together with tosilizumab (8 mg/kg, i.v.). Blood gas analysis showed PaO_2_ 63 mmHg, and SaO_2_ 92.2% (oxygenation index: 157.5) (Figure 2). At this point, it was evident that the patient's respiratory damage was potentially life‐threatening, and his CIP level was classified as 4. After three days of hospitalization, taking into account that the CIP of this patient was of the steroid‐resistant type, tacrolimus was added. Following another two weeks of treatment, the patient's shortness of breath had not significantly improved, and blood gas analysis showed PaO_2_ 107 mmHg, SaO_2_ 98% (IP 12cmH_2_O, EP 5cmH_2_O，FiO_2_ 50%, oxygenation index: 214). We readministered tosilizumab, but re‐examination of his chest CT still showed no significant improvement in interstitial inflammation (the right lung was slightly improved while the left lung was further aggravated) (Figure 1(d)).

Considering there was still no significant change on chest CT, and the residual lesions were mainly fibrotic, we believed the patient's condition to be less reversible. Intravenous methylprednisolone was replaced with oral prednisone and pirfenidone was added for antifibrotic treatment. To our surprise, the addition of pirfenidone significantly reduced the patient's severe dyspnea, and we were thus able to gradually reduce his oxygen intake concentration until he was switched to a normal double‐nasal catheter (5 l/min). The patient felt his mental condition subsequently improved and a lower oxygen supplement was well tolerated. Re‐examination of blood gas analysis showed PaO_2_ 94 mmHg and SaO_2_ 97.6% (oxygenation index: 229). As shortness of breath was still evident, the patient was discharged home on oxygen therapy and asked to continue taking prednisone, tacrolimus, and pirfenidone. We subsequently learned from follow‐up that the patient's dyspnea had worsened three months after discharge and tumor cells had been found in a pleural effusion sample. Eventually, the patient died of tumor progression.

## DISCUSSION

As far as we are aware, this is the first case report on the treatment of refractory CIP with pirfenidone. The immune system, activated by immune checkpoint inhibitors, may attack normal tissues and organs, leading to irAEs, which in the lung are considered CIP. The incidence of CIP reported in the real world has been reported to range from 13%–19%.^4^, ^5^

Our patient developed new pulmonary infiltrates after the first course of treatment and his disease progressed rapidly, which is consistent with a report in an earlier study that CIP levels may be higher if they occur at an early stage of ICI treatment.^6^ Studies have shown that patients receiving PD‐1/PD‐L1 monoclonal antibody are more likely to develop pulmonary infection than those receiving chemotherapy or a placebo.^6^ Therefore, in this report, when pulmonary inflammation was first determined in this patient, we empirically and actively used broad‐spectrum antibiotics for anti‐infective treatment. The level of CIP was defined as 4 due to the patient's obvious dyspnea and low oxygenation index, which were life‐threatening.^7^ The use of ICIs should be suspended in patients clearly diagnosed with grade 2 or above CIP. The basic treatment for CIP is steroid therapy and it has been previously reported that regular and adequate steroid therapy can control CIP in 70%–80% of patients.^8^ However, for patients with steroid‐resistant CIP, there is, at present, no unified standard treatment modality. Common treatments previously reported include shock steroid therapy, intravenous immunoglobulin (IVIG), IL‐6 receptor inhibitors, TNF‐inhibitors, or immunosuppressants.^9^, ^10^, ^11^ In the case reported here, we immediately discontinued pembrolizumab and the patient was treated with medranone, IVIG, tacrolimus and noninvasive ventilator‐assisted ventilation. Due to the severe shortness of breath in our patient, high‐flow oxygen was also administered together with tosilizumab during hospitalization. However, these treatments had little effect on his dyspnea. In addition, chest CT showed almost no improvement after two weeks of combined treatment.

Pirfenidone is a pyridinone compound that can play a broad‐spectrum antifibrotic role by inhibiting collagen synthesis, whilst providing anti‐inflammatory and antioxidative effects.^12^ The efficacy of pirfenidone was a surprise in the patient in this study whose shortness of breath improved significantly with an increase in his oxygenation index. Finally, we successfully replaced high‐flow oxygen with normal double‐nasal catheter oxygen, and the patient was discharged home on oxygen therapy. Unfortunately, the patient did not return for a further chest CT examination, so we were unable to obtain further imaging data.

According to the findings in our study, we consider that pirfenidone may be helpful in the treatment of steroid‐resistance CIP, and further clinical studies should be considered to explore its exact efficacy.

## CONFLICT OF INTEREST

The authors have no conflicts of interest to declare.
